# Analyzing the coupling characteristics of real-time key parameters in the thermal runaway of NCM523 batteries

**DOI:** 10.1016/j.isci.2026.114913

**Published:** 2026-02-05

**Authors:** Chen Zhong, Yi-Jing Gao, Li-Feng Zhou, Kai Liu, Li-Ying Liu, Hong-Ming Na, Yi-Song Wang, Tao Du

**Affiliations:** 1 Shenyang Fire Science and Technology Research Institute of EME, Shenyang 110034, China; 2 Key Laboratory of Eco-Industry, Ministry of Ecology and Environment, School of Metallurgy, Northeastern University, Shenyang 110819, China

**Keywords:** Energy systems, Thermal engineering, Energy storage

## Abstract

Lithium-ion batteries (LIBs) based on LiNi_0.5_Co_0.2_Mn_0.3_O_2_ (NCM523) are widely used in electric vehicles, yet thermal runaway (TR) remains a critical safety challenge. In real battery packs, materials filled between batteries reduce heat transfer during TR, frequently inducing a low-power heating phenomenon that is prevalent yet not fully understood, hindering the development of effective TR suppression and hazard mitigation strategies. Herein, this work investigates NCM523 LIBs’ TR characteristics via low-power heating experiments, delineating TR into four stages and identifying key gaseous products. Deflagration is mainly triggered by CO and H_2_ from polyvinylidene fluoride decomposition at 260°C, with flame behaviors strongly dependent on state of charge (SOC): high-SOC (>60%) batteries show significant deflagration, while low-SOC ones exhibit variable combustion duration and larger residual mass fluctuation. This work clarifies the gas-flame coupling mechanisms, addresses existing research deficiencies, and provides theoretical support for NCM523 LIB safety design and fire-suppression optimization.

## Highlights

• Accurate internal temperature assessment before battery thermal runaway (TR)
• Analysis of the mechanism of TR induced by low-heating
• Real-time smoke and gas characteristics during the TR process

## Graphical abstract

**Figure:**
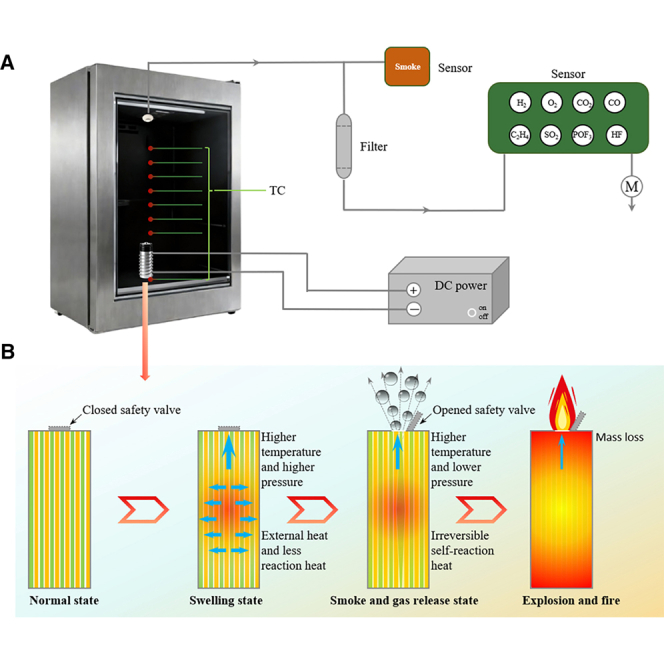

## Introduction

Lithium-ion batteries (LIBs) stand as one of the most pivotal energy technologies, yet their safety concerns primarily stem from mechanical, electrical, and thermal abuse, with thermal runaway (TR) being the ultimate hazardous manifestation.^1^^,^^2^^,^^3^ Potential manufacturing defects during production processes may induce battery malfunctions, while extreme ambient temperatures during service also serve as critical triggers for fault occurrence. Fault-induced runaway can further provoke electrochemical and chemical reactions of battery materials, culminating in TR accompanied by characteristic responses including smoke emission, ignition, gas release, and abrupt temperature elevation.^4^^,^^5^^,^^6^ At this stage, a cascade of uncontrolled exothermic chemical reactions leads to rapid temperature surges and massive gas generation, thereby triggering gas explosions and escalating fire risks.^7^ In medium-to-large-scale multi-cell battery systems, such as electric vehicle traction batteries, TR tends to propagate from a single battery cell to adjacent units, exacerbating the overall hazardous scenario. Given the flammability and toxicity of substances generated during LIB TR, investigating the accompanying characteristic behaviors throughout the TR process is of paramount significance.^8^^,^^9^

The fire behavior of LIBs following TR is a complex multi-stage process driven by chemical reactions, material compositions, mechanical abuse, and environmental conditions.^10^^,^^11^ It is crucial to distinguish between two related yet distinct events: the activation of the safety valve and the onset of TR.^12^^,^^13^ Prior to these stages, the decomposition of the solid electrolyte interface (SEI) film, reactions between the electrodes and electrolyte, and electrolyte degradation occur at an approximate temperature range of 90°C–120°C, followed by the intercalated lithium to react with the electrolyte.^14^ It can occur across different states of charge (SOC), but the risk is significantly higher under high SOC conditions.^15^ Upon safety valve activation, gas emission initiates but does not necessarily lead to combustion. This early stage typically involves the release of carbonate-based gases, a phenomenon consistently observed across different battery chemistries and SOC levels. When TR is triggered, the internal temperature and pressure of the battery rise sharply, initiating a second, more intense gas release stage characterized by the forceful ejection of high-temperature gases, aerosols, and solid particles.^16^^,^^17^ The emitted gas mixture, including hydrogen (H_2_), carbonic oxide (CO), carbon dioxide (CO_2_), and low-molecular-weight hydrocarbons, depends on the cathode chemical composition and gas constituents.^6^ LIB fires typically exhibit the characteristics of partially premixed jet flames, with white sparks ejected without visible flames in some instances and jet flames forming instantaneously in others.^18^ In conventional battery TR studies, high-power heaters with output powers of 300 W or even 1000 W are frequently employed to guarantee the deterministic initiation of battery TR,^19^ yet the direct damage to the battery housing caused by excessively high heating power, and the deviation from actual application scenarios are often overlooked because in real battery packs materials filled between batteries reduce heat transfer during TR, frequently inducing a low-power heating phenomenon. And for the vast majority of application scenarios, thermal runaway is usually caused by the accumulation of heat within the system itself.^20^^,^^21^ Furthermore, while extensive research has been conducted on temperature, smoke, gaseous products, and flame during battery TR, their real-time coupling effects have often been overlooked.^22^ Deciphering these characteristic behaviors holds significant implications for battery safety research and fire rescue operations.

Herein, this work focuses on investigating the TR characteristics of commercial LIBs, with particular emphasis on the NCM523-based LIBs. As a widely used commercial battery in electric vehicles (EVs), exploring its TR boundary conditions via low-power heating is crucial, given that its TR boundary conditions and characteristic behaviors are of paramount importance for battery safety and fire emergency response. Key research focuses include the dynamic evolutions of smoke, flame, gas, and temperature, coupled with the real-time battery status, aiming to reveal the real-time variations of smoke and gas during different TR stages. Finally, combined with the analysis of internal battery components, the fundamental mechanisms underlying the changes in gas and flame during the TR process will be elucidated. This study provides valuable guidance for the safe application of LIBs and the development of fire-fighting measures following TR.

## Results and discussion

### Research illustration

**Figure 1:**
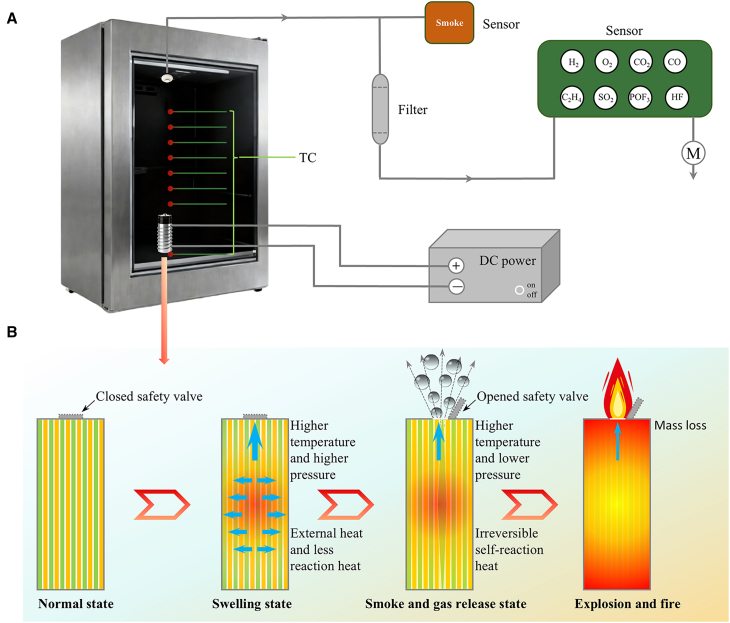
Test device and schematic diagram of battery thermal runaway process (A) Self-designed battery thermal runaway test apparatus including firebox, temperature measurement system, gas monitoring system and heating system. (B) Schematic of the thermal runaway process for NCM523 batteries.

In this work, TR-related information throughout the entire process will be monitored, including the external battery temperature, flame status, the types and concentrations of released gases, and so forth. The results will be analyzed to elucidate the states and underlying mechanisms of the battery during the TR process. To replicate TR phenomenon of batteries under real-world application scenarios and conduct relevant characteristic research, as shown in Figure 1A, a low-power 30 W heating wire was uniformly wound around the battery in this study. A thermal conductive film was placed between the battery sidewall and the heating wire to ensure uniform heating, with a DC power supply used as the heating source. The battery tests were performed in a sealed, atmospheric-pressure chamber equipped with thermocouples, smoke sensors, and gas sensors. Prior to entering the gas sensors, the gas was filtered through a filter. The gas sensors were specifically configured to detect H_2_, O_2_, CO_2_, CO, C_2_H_4_, SO_2_, POF_3_, and HF. A schematic diagram of the battery TR process is illustrated in Figure 1B. Overall, the process can be divided into four distinct stages: the normal state, swelling state, smoke and gas release state, and explosion and fire state. In the normal state, the battery exhibits detectable electrochemical characteristics, based on which the internal temperature of the battery prior to TR will be estimated. During the swelling stage, the battery sustains irreversible damage and loses its electrochemical functionality, accompanied by a rise in internal pressure. When the internal pressure reaches the activation threshold of the safety valve, the valve opens, and the battery enters the smoke and gas release stage. Heat continues to accumulate inside the battery in this stage, and once the temperature reaches the ignition point of the flammable gases released by the battery, deflagration occurs with jet-like flame ejection. Then the flame persists until all combustible substances inside the battery are fully consumed.

### Internal-external temperature coupling

**Figure 2:**
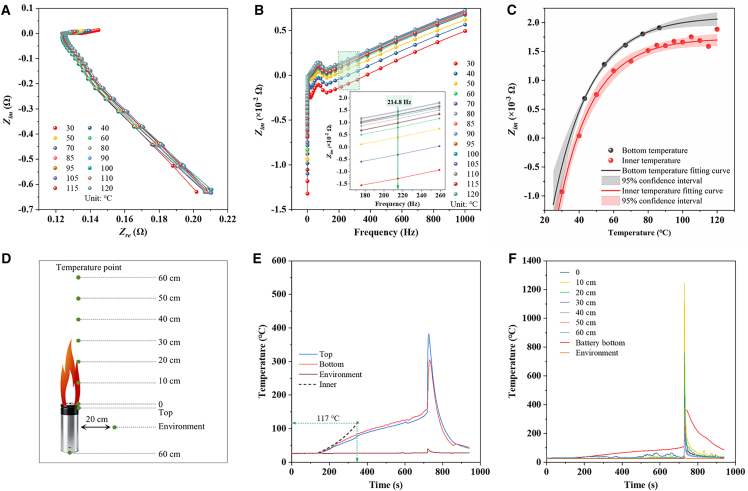
Temperature monitoring during the battery thermal runaway process (A) AC impedance spectra under different constant temperatures. (B) Imaginary parts of impedance from 10 kHz to 0.1 Hz at different temperatures. The inset is imaginary parts at 214.8 Hz. (C) The corresponding relationship between the bottom temperature and the inner temperature before battery failure. (D) Schematic diagram of spatial temperature monitoring during battery thermal runaway process. (E) Battery temperature monitoring curve during the battery thermal runaway process. (F) Temperature monitoring curve of the battery surrounding space during thermal runaway. Data are represented as mean ± standard deviation.

Once the TR temperature is reached, the battery will undergo TR. Therefore, investigating the critical temperature is essential. However, merely measuring the battery’s surface temperature is insufficient. To address this, the coupling of the internal and external temperatures of the battery is achieved based on EIS technology. The preset test temperature range is 30°C–120°C, with additional key temperature points incorporated. Figure 2A presents the EIS curves, measured over a frequency range of 0.1–1000 Hz. By decomposing the real part, imaginary part, and phase angle, the relationship between the imaginary part of impedance (Z_im_) and frequency is obtained, as shown in Figure 2B. Through comparing the distribution patterns of Z_im_ at different frequencies, the relationship between Z_im_ and temperature at 214.8 Hz is identified as suitable for data analysis and fitting. As illustrated in Figure S1 and Table S1, a cubic polynomial fitting is determined to be the optimal method via comparative analysis of data fitting results, with a coefficient of determination (R^2^) of 97.41, which meets the statistical requirement of a 95% confidence interval. Although the R^2^ of quartic polynomial fitting (R^2^ = 97.79) is slightly higher than that of cubic polynomial fitting, cubic polynomial fitting is deemed more compliant with data statistical requirements, considering factors such as data convergence rate. A relationship between Z_im_ and temperature was established. To couple the bottom temperature and inner temperature, the alignment was based on the consistency of the Z_im_ value, as illustrated in Figure 2C. When the internal temperature approached 120°C, the points exhibited a tendency to deviate from the 95% confidence interval, indicating the failure of its electrochemical functionality, with the corresponding bottom temperature being approximately 75°C. Thus, for its applicability boundary after electrochemical failure, rigorous validation experiments were conducted. When the temperature at the bottom of the battery casing reached more than 72°C (with the corresponding internal temperature at 117°C), the probability of data scattering within the 95% confidence interval increased significantly. The failure boundary is indicated by green arrows in Figure 2C.

### Phenomenon of thermal runaway

To further explore the battery’s failure temperature and investigate the characteristics from battery failure to deflagration, thermocouples (TCs) were installed at different sites, the bottom and top of the battery, and 5 cm away from the flame axis above the battery (Figure 2D). Meanwhile, an additional TC was placed 20 cm away from the middle of the battery to monitor the environmental temperature change during the TR process. The battery temperature monitoring results are presented in Figure 2E. The battery with the 100% SOC started to be heated at 150 s, and the bottom and top temperatures were nearly identical before 220 s. At approximately 340 s, the electrochemical characteristics disappeared, with the bottom temperature reaching around 75°C and the top temperature around 80°C. The temperature difference was attributed to the convection of the electrolyte inside, with the internal temperature being approximately 117°C at this stage. As heating continued, the battery’s safety valve activated at around 600 s. With the battery pressure relief, the bottom temperature decreased significantly, while the top temperature showed no obvious downward trend due to the influence of high-temperature gases, leading to a further increase in the temperature difference between them. At 740 s, the battery underwent deflagration accompanied by jet-like flames, resulting in a sharp surge in both the bottom and top temperatures, which reached approximately 300°C and 390°C, respectively. After deflagration, little combustible material remained inside the battery casing, and the flame persisted briefly before extinguishing, followed by a rapid drop in the battery casing temperature. TC results of the battery casing and along the flame path above the battery are presented in Figure 2F. Prior to deflagration, only the TC positioned at 0 cm above the battery exhibited a significant fluctuating upward trend, while the other thermocouples were minimally affected by thermal radiation. After deflagration, the temperature at the 10 cm location surged to over 1200°C, corresponding to the core temperature of the deflagration flame, and the temperature at 20 cm reached approximately 800°C. Since the TC data for 50 cm and 60 cm are quite similar, it is believed that the actual height of the flame is approximately between 50 cm and 60 cm. Due to thermal inertia, the temperature of the battery casing gradually decreased following flame extinction.

**Figure 3:**
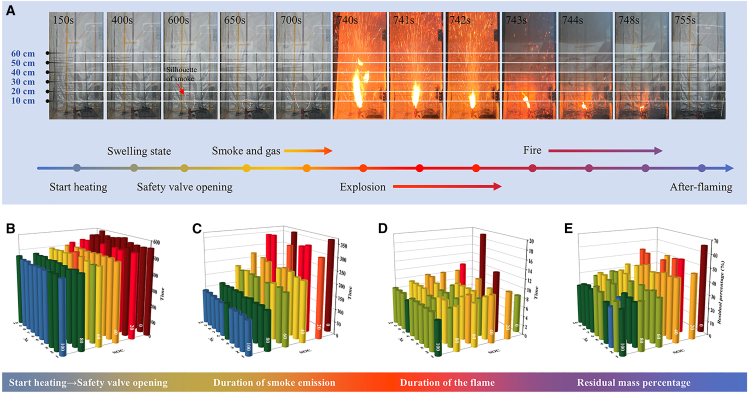
The thermal runaway process |(A) The images of the thermal runaway process for the 100% SOC batteries. (B) The duration comparison from start heating to safety valve opening for the batteries with different SOC. (C) The duration comparison from safety valve opening to explosion for the batteries with different SOC. (D) The duration comparison of the flame for the batteries with different SOC. (E) The residual mass percentage comparison after flaming for the batteries with different SOC. Data are represented as mean ± standard deviation.

Testing images of the thermal runaway process for the 100% SOC batteries are presented in Figure 3A. From the initiation of heating, the battery underwent a swelling stage until the safety valve activated, at which point a large volume of smoke and gas was ejected instantaneously. Subsequently, after a brief period of attenuation, the smoke and gas emissions maintained a stable and substantial diffusion. At approximately 740 s, the flammable gases and substances were ignited alongside jet flames. The flame ejection persisted for 2 s, after which the flame intensity diminished and transitioned to a free-burning flame configuration. The flame was extinguished at 748 s. To intuitively compare TR processes of batteries with different SOC (0%, 20%, 40%, 60%, 80%, and 100%), statistical data on phenomena observed at various stages from heating to after-flaming are presented in Figures 3B–3E, with 10 batteries tested for each SOC, where the color coding directly corresponds to the determinative data associated with specific SOC levels. As shown in Figure 3B, the time elapsed from heating initiation to safety valve activation increased significantly with decreasing SOC, rising from an average of approximately 450 s–550 s. However, for low-SOC batteries, a large proportion failed to ignite after smoking (Figure 3C), accompanied by a marked increase in smoke duration and a reduction in ignition probability. As illustrated in Figure 3D, batteries with SOC above 60% exhibited prominent deflagration, accompanied by the ejection of substantial battery materials, and the flame duration generally ranged from 6 s to 8 s. In contrast, for low-SOC batteries, after mild deflagration, some batteries sustained combustion for nearly 20 s while others extinguished shortly after ignition, which is attributed to variations in air contact extent. Due to the low deflagration intensity, a large amount of combustible material remained inside the batteries, supporting sustained combustion; conversely, oxygen supply was insufficient during combustion due to the safety valve restricting oxygen ingress. Influenced by deflagration intensity, the residual mass percentage of batteries varied significantly (Figure 3E). For high-SOC batteries, the residual mass percentage was primarily distributed around 30%, whereas for low-SOC batteries, it exhibited greater variability due to the significant impact of combustion duration.

### Process and mechanism of thermal runaway

**Figure 4:**
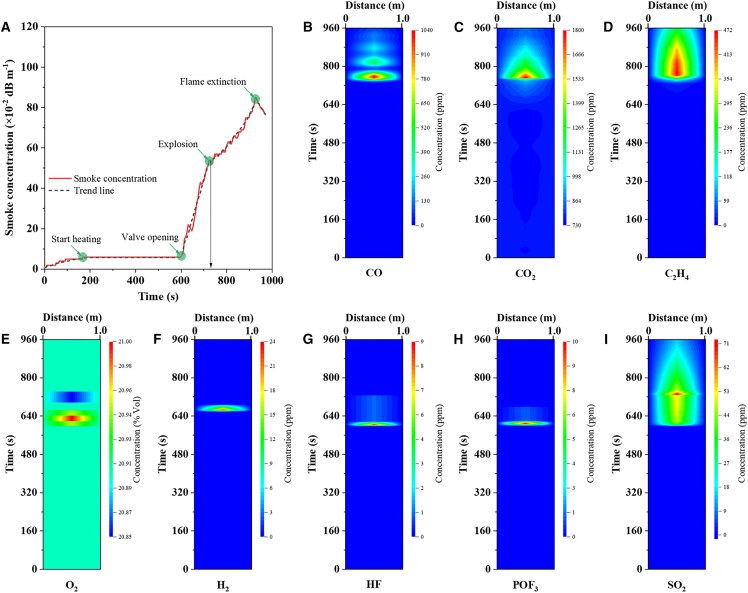
Monitoring of gas emission during the process of the battery thermal runaway (A) Smoke monitoring curve during the thermal runaway process. (B–I) Gas monitoring curve during the thermal runaway process for (B) CO, (C) CO_2_, (D) C_2_H_4_, (E) O_2_, (F) H_2_, (G) HF, (H) POF_3_, and (I) SO_2_. Data are represented as mean ± standard deviation.

The release of smoke and gases is the most critical characteristic to monitor during TR and constitutes the fundamental factor influencing battery safety. Real-time monitoring data of smoke and gas release, acquired via sensors installed far from 1.5 m along the battery axis, are presented in Figure 4. Figure 4A illustrates the dynamic changes in smoke concentration throughout the entire TR process, from the initiation of heating to flame extinction, which corroborates the division of the four TR stages as described in Figure 1. The timing of heating initiation was determined based on the stabilization of the smoke sensor. Due to the lag associated with smoke accumulation and diffusion, smoke concentration monitoring exhibited a slight delay compared to temperature monitoring. Upon the activation of the safety valve, a large volume of smoke was rapidly ejected, with the smoke concentration exceeding 0.2 Db·m^−1^. However, influenced by the reduction in smoke emission and diffusion in the large testing space, the smoke sensor reading displayed a transient decrease at approximately 640 s. Subsequently, substantial smoke escaped, and the concentration surged to 0.52 Db·m^−1^, at which point the battery underwent deflagration. The upward trend of smoke concentration slowed with the emergence of the flame, peaking at 0.87 Db·m^−1^ before declining as the flame extinguished.

CO, CO_2_, and C_2_H_4_ are carbon-containing characteristic gases, primarily derived from the thermal decomposition and combustion of electrolyte solvents. As illustrated in Figure 4B, CO was first detected at 740 s and rapidly accumulated to a peak exceeding 1000 ppm at 750 s, with the battery casing temperature reaching approximately 300°C. Concurrently, the battery underwent deflagration, indicating that the rapid accumulation of CO is one of the critical factors inducing battery deflagration. Following the termination of deflagration, the CO concentration experienced a transient decrease, and subsequently re-accumulated in the space due to the incomplete combustion of the electrolyte and conductive agents within the battery. The CO concentration ultimately diminished via diffusion only after flaming. In Figure 4C, CO_2_ emission commenced immediately upon the activation of the safety valve, with a concentration peak of 1800 ppm observed post-deflagration. This peak is attributed to the combined effects of CO_2_ released from thermal decomposition and CO combustion. The CO_2_ concentration decreased significantly once the battery fire had ended. Figure 4D presents the concentration variation of ethylene (C_2_H_4_) after the safety valve opened, approximately 5 ppm of C_2_H_4_ was continuously emitted. Upon battery deflagration, the C_2_H_4_ concentration surged instantaneously to over 470 ppm, followed by a gradual decline due to gas diffusion.

O_2_ and H_2_ are primarily attributed to the decomposition and combustion of electrode materials. Upon the activation of the safety valve, with the release of lattice oxygen from NCM523, the content of O_2_ in the box space increased slightly from 20.93% to 21% due to gas accumulation. After deflagration, it decreased significantly to approximately 20.85%. H_2_ was first detected at around 660 s, corresponding to the middle stage of gas release, during which the battery temperature rose sharply but deflagration had not yet occurred. Hydrogen fluoride (HF) and phosphorus oxyfluoride (POF_3_) were completely emitted in the early stage following safety valve activation, with their concentrations gradually decreasing via diffusion. These gases are considered degradation products of battery additives. Sulfur dioxide (SO_2_) exhibited a significant emission upon safety valve activation and a sharp surge during deflagration, which is attributed to the combined decomposition of by-products generated during the reactions between the electrolyte and battery components.

**Figure 5:**
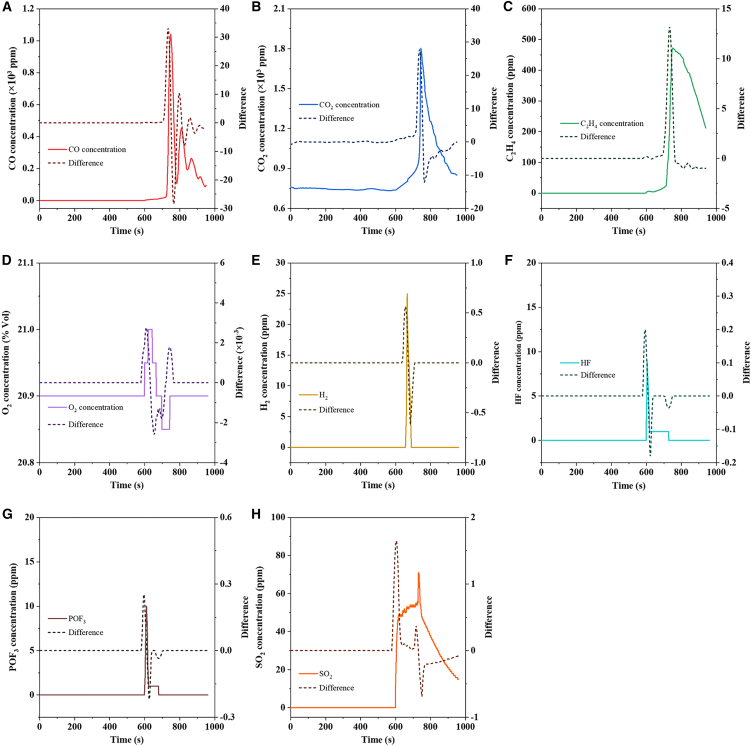
Dynamic analysis of gas emission during the process of thermal runaway (A–H) Gas concentration and differential curve for (A) CO, (B) CO_2_, (C) C_2_H_4_, (D) O_2_, (E) H_2_, (F) HF, (G) POF_3_, and (H) SO_2_. Data are represented as mean ± standard deviation.

To more clearly elucidate the gas release rates at different time points, time-based differential calculation was applied to the gas release curves (Figure 5). In the initial stage of battery deflagration, CO, CO_2_, and C_2_H_4_ exhibited similar release rate trends, which are believed to be primarily driven by high temperatures (Figures 5A–5C). After the safety valve activation, the battery achieved self-heating, with the temperature rapidly rising to approximately 300°C within a short period, enabling the rapid decomposition of the electrolyte. With the occurrence of battery deflagration, CO underwent several concentration fluctuations due to incomplete combustion. Given the heating effects on gas dynamics, only trend analysis is performed for the O_2_ volume fraction acquired from the sensor. O_2_ mainly experienced three stages, including lattice oxygen release, oxygen consumption during deflagration, and diffusion replenishment (Figure 5D), thus showing a release rate trend of initial increase, subsequent decrease, and then re-increase.^23^ As illustrated in Figure 5E, trace amounts of H_2_ are reaction products of lithium, while the main H_2_ evolution is derived from the decomposition of polyvinylidene fluoride (PVDF) at high temperatures.^24^^,^^25^ When the temperature reached 260°C, a large amount of H_2_ was generated in a short time, inducing deflagration. The release rate trends of HF and POF_3_ were completely consistent (Figures 5F and 5G), given their identical sources and formation conditions during the internal heat accumulation stage prior to safety valve activation.^26^^,^^27^^,^^28^ After release, due to their non-flammable and corrosive properties, they underwent spatial transfer via diffusion, and the sensor continuously displayed low-limit value alerts. Figure 5H presents the differential curve of the SO_2_ release rate. A significant rate increase was observed in the early stage of safety valve activation, and a secondary peak emerged during deflagration with the intensification of side reactions. For a more comprehensive and detailed comparison, the gas release characteristics of NCM523 batteries across various SOC levels during TR are summarized in Table S2, where the data indicate that decreasing SOC leads to slower and less complete TR reactions, thereby resulting in peak latency and a reduction in peak concentration.

**Figure 6:**
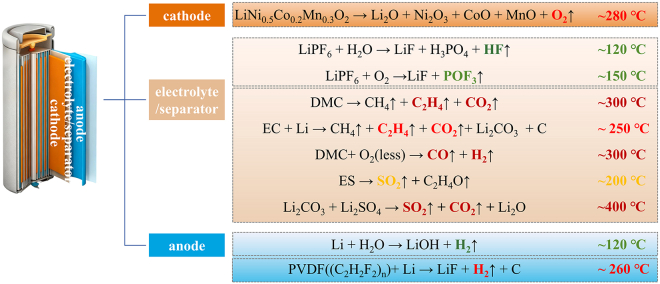
Analysis of the mechanism of battery thermal runaway

Leveraging the multi-parameter dataset encompassing gas concentration, smoke density, and flame behavior, which was acquired through a real-time, synchronous acquisition protocol for battery TR processes, the inherent coupling relationships among these parameters were concurrently captured during data collection. A systematic analysis was subsequently conducted by integrating gas release characteristics with TC measurements, on the basis of which the gases associated with battery TR were categorized. The corresponding classification results are presented in Figure 6. Prior to safety valve activation, electrolyte decomposition and accumulation of HF and POF_3_ occurred, and a certain amount of H_2_, derived from the reaction of deposited Li, was generated.^19^^,^^29^^,^^30^^,^^31^ With the activation of the safety valve and the occurrence of further TR, self-heating led to a rapid rise in the internal temperature, resulting in the decomposition of NCM523 and the emission of carbon-based gases. Finally, affected by combustion, the external temperature of the battery exceeded 400°C, and sulfur in the by-product lithium sulfate (Li_2_SO_4_) was further released.^32^^,^^33^ Given that battery TR arises from the combined effects of external heating and self-generated heat, strengthening the monitoring function of the battery management system facilitates the early detection of battery malfunctions, as well as the execution of forced cooling and flammable gas exhaust protocols, which serve as an effective strategy to alleviate TR-related risks.

### Limitations of the study

The NCM523 LIBs tested in this work are 18650-type cells equipped with a safety valve. The characteristics monitored during testing, including smoke, gas, and temperature, are closely associated with the battery’s chemistry and model. LIBs with different specifications or structural variations will exhibit distinct TR behaviors under the same test conditions. While the safety valve primarily functions to relieve pressure, it also guides the ejection direction of smoke and gas. Notably, the type of safety valve also plays a crucial role in analogous studies, indicating potential variability in results across different valve designs.

## Resource availability

### Lead contact

Requests for further information and resources should be directed to and will be fulfilled by the lead contact, Li-Feng Zhou (zhoulf@smm.neu.edu.cn).

### Materials availability

This study did not generate new unique reagents.

### Data and code availability

• The article includes all data generated or analyzed during this study. Detailed experimental procedures and data are provided in the methods and supplemental information.
• No code was generated in this study.
• Any additional information required to reanalyze the data reported in this article is available from the lead contact upon request.

## Acknowledgments

C. Zhong, Y. Gao, and L. Zhou made an equal contribution to this work. This work was supported by the National Standard Plan Project (No. 20100090-Q-312), the 10.13039/501100001809National Natural Science Foundation of China (No. U24A20195 and No. 52270177), the Liaoning Province Science and Technology Plan Joint Program (Key Research and Development Program Project) (2023JH2/101800058), Postdoctoral Fund of 10.13039/501100004184Northeastern University (20240204), the Fundamental Research Funds for the Central Universities (No. N2425035) and the Doctoral Research Initiation Fund Program in Liaoning Province (2025-BS-0126).

## Author contributions

Conceptualization, L.-F.Z.; methodology, C.Z., L.-F.Z., Y.-J.G., L.-Y.L., and Y.-S. W.; investigation, L.-F.Z. and Y.-J.G.; writing – original draft, C.Z., L.-F.Z., and Y.- J.G.; writing – review and editing, T.D., H.-M.N., and K.L.; funding acquisition, L.-F.Z. and T.D.; resources, L.-F.Z. and C.Z.; supervision, T.D. and L.-F.Z.

## Declaration of interests

The authors declare no competing interests.

## STAR★Methods

### Key resources table

**Table:** 

REAGENT or RESOURCE	SOURCE	IDENTIFIER
**Deposited data**		

Smoke and gas changes	This work	N/A
Temperature trend	This work	N/A

**Software and algorithms**		

Origin 2018	OriginLab corporation	ID: 0IE-J22-ACM
Gas Monitoring	Qianzhao Tech.	N/A

**Other**		

Gas sensors	Qianzhao Tech.	N/A

### Method details

#### Battery parameter

The lithium-ion battery samples employed in this work are 18650 cylindrical cells with a nominal capacity of 3.2 Ah, which were unmarked commercial standard cells of the same batch, customized by a leading manufacturer in the battery industry. The cathode material is LiNi_0.5_Co_0.2_Mn_0.3_O_2_ (NCM523), and the anode is composed of graphite. The electrolyte consists of a solution of LiPF_6_ dissolved in a mixture of ethylene carbonate (EC) and dimethyl carbonate (DMC), with ethylene sulfite (ES) added as an additive. Each battery has a mass of 48 g and dimensions of 18.35 × 65.00 mm (diameter × height). To ensure the consistency of the initial state of charge (SOC) across all test samples, each battery was subjected to three pre-test cycles, charging at 0.5 C via constant current-constant voltage (CC-CV) mode and discharging at 0.1 C also via CC-CV mode. A total of 60 batteries were utilized in the regular experiments, with 10 units tested for each SOC level, which was determined via the coulomb counting method.

#### Heating parameter

Heating power is a pivotal parameter that directly governs the reliability and validity of battery TR experiments. To ensure the heating condition aligns with practical scenarios, this parameter was rigorously optimized through systematic pre-experiments, where batteries with 40% SOC were selected as model samples. Uniformly configured heating wire was adopted as the heating apparatus, with the input power stepwise incremented from an initial value of 10 W. Throughout the calibration process, the TR behaviors of the test batteries were continuously monitored. Specifically, when the power reached 30 W with the temperature rise rate of less than 0.3 °C s^-1^ before TR based on the bottom temperature, a reproducible probability of flame emergence during the TR progression was stably detected, and this power was therefore designated as the fixed heating power for all subsequent formal experiments.

#### Experimental apparatus and gas collection

To ensure the unobstructed ejection of flames and free diffusion of gases generated during the test, all experiments were conducted in a sealed rectangular steel chamber with dimensions of 1000×1000×2000 mm (length×width×height). Prior to each test, the chamber was subjected to three pressurized airtightness tests to prevent gas leakage during the experimental process. Specifically, the test criteria are as follows, 10 minutes after filling with nitrogen, the change in the gas composition inside the box was less than 1%. The gas sensors were calibrated and verified using standard gases. After three replicate tests, the maximum allowable measurement error was set to ±5%. Temperature measurements were performed with K-type thermocouples (TC) (accuracy: ±0.1°C) at a sampling interval of 1 second. Flame ejection behavior was recorded using a high-speed camera with a frame rate of 120 fps.

#### Smoke and gas characteristics

The concentration characteristics of smoke and gaseous products during battery TR were quantified using chemistry-based gas sensors positioned 1.5 m above the battery top, with the sensor installation location tailored to standard battery packaging configurations and potential gas exhaust directions. The target analytes recognized by the sensors include smoke, CO, CO_2_, and C_2_H_4_, O_2_, H_2_, HF, POF_3_, SO_2_, which are connected in parallel to achieve synchronous signal acquisition. All sensors were calibrated prior to experimental measurements following a standardized protocol: first, a clean background gas was introduced into the sensor chamber, and the stable output value was recorded as the zero-concentration reference. Subsequently, high-purity standard gases (99.999%) with gradient concentrations (from low to high) were sequentially injected into the sensor system. For each concentration gradient, the gas supply was maintained at a steady state for 30 minutes, and the corresponding output signal values were recorded. The “standard gas concentration-sensor output value” dataset was compiled, and a calibration equation was established via linear regression analysis. To ensure measurement reliability, 1-2 intermediate-concentration standard gases were randomly selected for replicate tests (2-3 cycles), with the output deviation required to be within the acceptable range (≤±1%). To avoid the issues of sensor cross-sensitivity, the validation was performed by introducing gas mixtures with fixed proportions and comparing the sensor output data with pre-calibrated reference values, and the errors of the target gases are all below 2%.

To conclude, the TR characteristics of NCM523-based LIBs were systematically explored via low-power heating experiments. The TR process was delineated into four distinct stages: normal operation, swelling, smoke/gas emission, and explosion/combustion. Key gaseous products identified in this study are H_2_, O_2_, CO_2_, CO, C_2_H_4_, SO_2_, POF_3_, and HF. Deflagration is primarily triggered by the rapid accumulation of CO (peak concentration > 1000 ppm) and H_2_, which are derived from the thermal decomposition of PVDF at 260 °C. CO_2_ with the peak concentration of 1800 ppm, originates from both the thermal decomposition of battery components and the combustion of CO, whereas the C_2_H_4_ concentration surges to >470 ppm upon deflagration. Flame behaviors are strongly dependent on the SOC, when batteries with SOC >60% exhibit prominent deflagration, characterized by a flame duration of 6-8 s, a flame height of 50-60 cm, and a flame core temperature exceeding 1200 °C, whereas low-SOC batteries display variable combustion durations and greater residual mass fluctuations. Collectively, these findings elucidate the intrinsic gas-flame coupling mechanisms during the TR of NCM523-based LIBs, and provide theoretical support for the safety design of LIBs and the optimization of fire-suppression strategies.

### Quantification and statistical analysis

#### Method for estimating internal temperature of batteries

The internal temperature of the battery is estimated based on electrochemical impedance spectroscopy (EIS).^34^ EIS was employed to characterize the electrochemical behavior of the battery, enabling in-depth analysis of its internal processes, such as charge-transfer resistance, diffusion properties, etc.^35^^,^^36^ Given the distinct time constants of these internal processes, a broad range of applied alternating current (AC) frequencies was adjusted to achieve targeted examination. In the measurement, a sinusoidal signal (either voltage or current) was injected into the battery at a specific frequency, and the resulting response signal, which is dependent on battery impedance, was recorded and analyzed. Galvanostatic EIS was selected as the preferred mode for LIBs testing, and the impedance was subsequently calculated using the following equation:

$$Z=\frac{V_{t}}{I_{t}}=Z_{0}\frac{\sin\left(\omega t\right)}{\sin\left(\omega t+\phi \right)}$$

Where *V*_*t*_ and *I*_*t*_ are the potential and current at time *t*, ∅ is the phase shift and *Z*_*0*_ is the magnitude of impedance. The impedance can then be expressed as a complex number comprised of both real (*Z*_*re*_) and imaginary (*Z*_*im*_) values using the following equations:

$$Z_{re}=Z_{0}\;\cos\left(\phi \right)$$

$$Z_{im}=Z_{0}\;\sin\left(\phi \right)$$

$$Z_{0}=\sqrt{{Z_{re}}^{2}+{Z_{im}}^{2}}$$

To quantify impedance variation with temperature, EIS measurements were conducted at 100% SOC over a temperature range of 30-120°C with 10°C increments, where the battery’s internal temperature was determined via prolonged standing in a constant temperature oven, eliminating the need for independent measurements. The average impedance at each frequency across the entire discharge step was calculated for model parameter fitting. After the battery is fully charged with the charging rate of 0.2C, since direct measurement of internal battery temperature is infeasible it should be left to stand in the constant temperature box for 3h to ensure that the internal and external temperatures of the battery are consistent. Polynomial fitting was applied to correlate the imaginary part of impedance at 214.8 Hz (dependent variable, y) with temperature (independent variable, x), with a separate calibration step performed for each individual battery.

$$y=Ax^{2}+Bx+C$$

It was found that the battery would lose its electrochemical properties when the inner temperature exceeded 120°C. And at temperatures below 120°C, the accuracy rate reaches 95%. In order to obtain the real-time internal temperature changes during the battery thermal runaway process, a coupling experiment between the bottom temperature of the battery and the internal temperature of the battery was conducted based on the consistency of EIS characteristics.
