# Supplementary material for: Analyzing the coupling characteristics of real-time key parameters in the thermal runaway of NCM523 batteries
Source: iScience. 2026 Feb 5;29(3):114913. doi: 10.1016/j.isci.2026.114913 (PMC12927304; doi:10.1016/j.isci.2026.114913)
Supplement: Document S1. Figure S1 and Tables S1 and S2 [file mmc1.pdf]

iScience, Volume *29*

Supplemental information

Analyzing the coupling characteristics of real-time key parameters in the thermal runaway of NCM523 batteries

Chen Zhong, Yi-Jing Gao, Li-Feng Zhou, Kai Liu, Li-Ying Liu, Hong-Ming Na, Yi-Song Wang, and Tao Du

Supporting information

Supplemental Figures

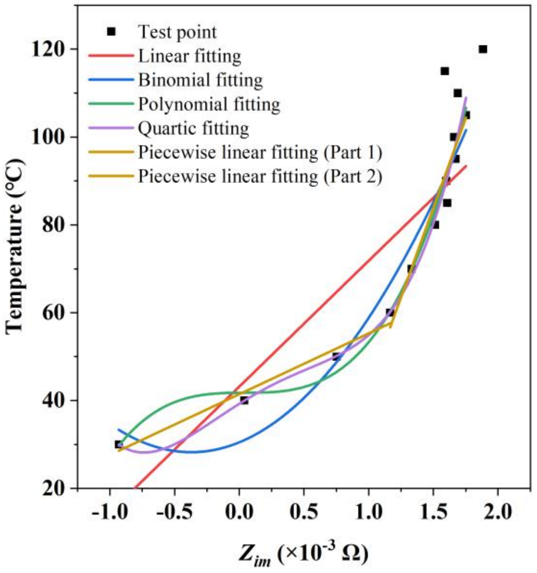

Figure S1 Comparison of fitting results.

Supplemental Tables

Table S1 Fitting parameters for $y=Intercept+B1x+B2x^2+B3x^3+B4x^4$ ($y=Z_{im}$, x=temperature)

Fitting types	Intercept	B1	B2	B3	B4	R^2
Linear fitting	43.18195	28650.06592	-	-	-	80.82
Binomial polynomial fitting	30.49719	12096.91316	1.62503E7	-	-	94.07
Cubic polynomial fitting	41.76308	226.46246	-1.60818E6	1.29147E10	-	97.41
Quartic polynomial fitting	39.26818	18516.4021	-5.6869E6	-6.90054E9	9.74471E12	97.79

Piecewise linear	41.45534	13837.93767	-	-	-	96.95
fitting	-38.32828	81472.81558	-	-	-	89.23

Table S2 Gas characteristics under different SOC states

SOC	Types of gases	Peak concentration	The time after the safety valve opens (s)
100	CO	1040 ppm	148
	CO_2	1803 ppm	144
	C_2H_4	470 ppm	155
	O_2	21 % Vol	26
	H_2	25 ppm	66
	HF	9 ppm	4
	POF_3	10 ppm	10
	SO_2	71 ppm	132
80	CO	1053 ppm	151
	CO_2	1655 ppm	146
	C_2H_4	461 ppm	157
	O_2	21 % Vol	27
	H_2	26 ppm	69
	HF	11 ppm	4
	POF_3	11 ppm	10
	SO_2	69 ppm	134
60	CO	1009 ppm	153
	CO_2	1649 ppm	148
	C_2H_4	455 ppm	160
	O_2	21 % Vol	27
	H_2	24 ppm	68
	HF	9 ppm	4

	POF_3	10 ppm	11
	SO_2	68 ppm	138
40	CO	898 ppm	160
	CO_2	1591 ppm	153
	C_2H_4	395 ppm	165
	O_2	21 % Vol	28
	H_2	21 ppm	72
	HF	8 ppm	5
	POF_3	8 ppm	12
	SO_2	66 ppm	145
